# miR-223 Regulates Cell Proliferation and Invasion via Targeting PDS5B in Pancreatic Cancer Cells

**DOI:** 10.1016/j.omtn.2019.01.009

**Published:** 2019-01-29

**Authors:** Jia Ma, Tong Cao, Yue Cui, Fan Zhang, Ying Shi, Jun Xia, Z. Peter Wang

**Affiliations:** 1 Department of Biochemistry and Molecular Biology, School of Laboratory Medicine, Bengbu Medical College, Anhui 233030, China; 2 Department of Clinical Laboratorial Examination, the First Affiliated Hospital of Bengbu Medical College, Bengbu, Anhui 233004, China; 3 Research Center of Clinical Laboratory Science, Bengbu Medical College, Anhui 233030, China; 4 Department of Pharmacology, Adagene Limited Company, Suzhou, Jiangsu 215000, China; 5 Department of Pathology, Beth Israel Deaconess Medical Center, Harvard Medical School, Boston, MA 02115, USA

**Keywords:** miR-223, PDS5B, growth, invasion, pancreatic cancer

## Abstract

Emerging evidence has demonstrated that miR-223 is critically involved in the progression of pancreatic cancer (PC); however, the underlying mechanisms are not fully elucidated. In the present study, we explored the molecular basis of miR-223-mediated tumor progression in PC. We performed numerous approaches including MTT, FACS, transfection, RT-PCR, western blotting, Transwell, and animal studies to determine the physiological role of miR-223 in PC cells. We found that sister chromatid cohesion protein PDS5 homolog B (PDS5B) is a direct target of miR-223 in PC. Moreover, PDS5B exhibits tumor-suppressive function in PC cells. Consistently, ectopic overexpression of PDS5B reversed miR-223-mediated tumor progression in PC cells. These results suggest that the miR-223/PDS5B axis regulates cell proliferation and invasion in PC cells. Our findings indicated that downregulation of miR-223 could be a novel therapeutic approach for PC.

## Introduction

Pancreatic cancer (PC) patients often have high morbidity and mortality. Having lethal malignant tumors, PC could lead to 44,330 deaths in the United States in 2018.^1^ It is expected that there will be 55,440 new cases of PC in the United States this year.^1^ In contrast to the steady increase in survival of most cancer types, the 5-year survival rate is 8% for PC patients, as these patients are typically diagnosed at a distant stage.^1^, ^2^ Another reason for the relatively low survival rate of PC is because of intrinsic and extrinsic drug resistance to chemotherapy.^3^, ^4^ Without a doubt, it is pivotal to explore the molecular mechanism of PC development and progression and develop a novel therapeutic strategy for the treatment of PC.

It has been reported that 5%–10% of PC development is due to a family history of this disease.^5^ Multiple studies have demonstrated that germline mutations are associated with PC, including BRCA2 (breast cancer 2), ATM (ataxia telangiectasia mutated), CDKN2A (cyclin-dependent kinase inhibitor 2A), and TP53 (tumor protein p53).^6^, ^7^ Several kinases such as BRAF (B-raf proto-oncogene, serine/threonine kinase), CDK6 (cyclin-dependent kinase 6), and mesenchymal-epithelial transition (MET) receptor tyrosine kinase have also been found to link to pancreatic tumorigenesis.^8^, ^9^ Emerging evidence has revealed that some signaling pathways are critically involved in pancreatic tumorigenesis, including WNT (wingless-type MMTV integration site), KRAS, MAPK (mitogen-activated protein kinase), Notch, and hedgehog.^10^, ^11^, ^12^ Recently, microRNAs (miRNAs) were identified to be involved in the development and progression of PC.^13^, ^14^, ^15^ For example, miR-223 has been investigated for its potential role in human cancers.^16^, ^17^ It has been reported that a high level of miR-223 is an independent factor for worse prognosis in pediatric lymphoblastic T cell lymphoma.^18^ Relative high expression of miR-223 was observed in sputum in non-small-cell lung cancer, suggesting that miR-223 could be a diagnostic useful biomarker for the detection of lung cancer.^19^ In gastric and esophageal cancers, miR-223 was upregulated in tissue samples and plasma of early-stage gastroesophageal adenocarcinoma.^20^ In oral cancer, circulating miR-223 might serve as a potential biomarker for diagnosis and therapeutic target for the treatment of cancer.^21^ Another independent study further supported the notion that plasma miR-223 might be a clinically useful biomarker for screening PC and predicting the invasiveness of PC.^22^ Consistently, compared with normal pancreas, miR-223 was overexpressed in intraductal papillary mucinous neoplasms, including PC tissues.^23^ Strikingly, urinary miR-223 was found to be a potential marker for early detection of PC, because miR-223 was significantly overexpressed in PC patients compared with age-matched healthy individuals.^24^ Although the role of miR-223 is investigated in PC, the molecular mechanism of miR-223-induced tumor progression is largely elusive.

PDS5B (precocious dissociation of sisters 5B), also known as APRIN, belongs to the PDS5 family. Recently, PDS5B was reported to be involved in DNA damage and carcinogenesis.^25^, ^26^ However, the function of PDS5B in PC has not been determined. In the present study, we investigated the role of PDS5B in PC cell growth, migration, and invasion. We also explored whether miR-223 targets PDS5B and exerts its oncogenic function in PC cells. Moreover, we determined the molecular mechanism of miR-223-triggered PC progression. We found that miR-223 exhibited its oncogenic function via targeting PDS5B in PC cells. Our study provided the evidence for targeting miR-223 as a novel therapeutic approach in PC.

## Results

### Overexpression of miR-223 Promotes Cell Growth and Inhibits Cell Apoptosis in PC Cells

**Figure 1:**
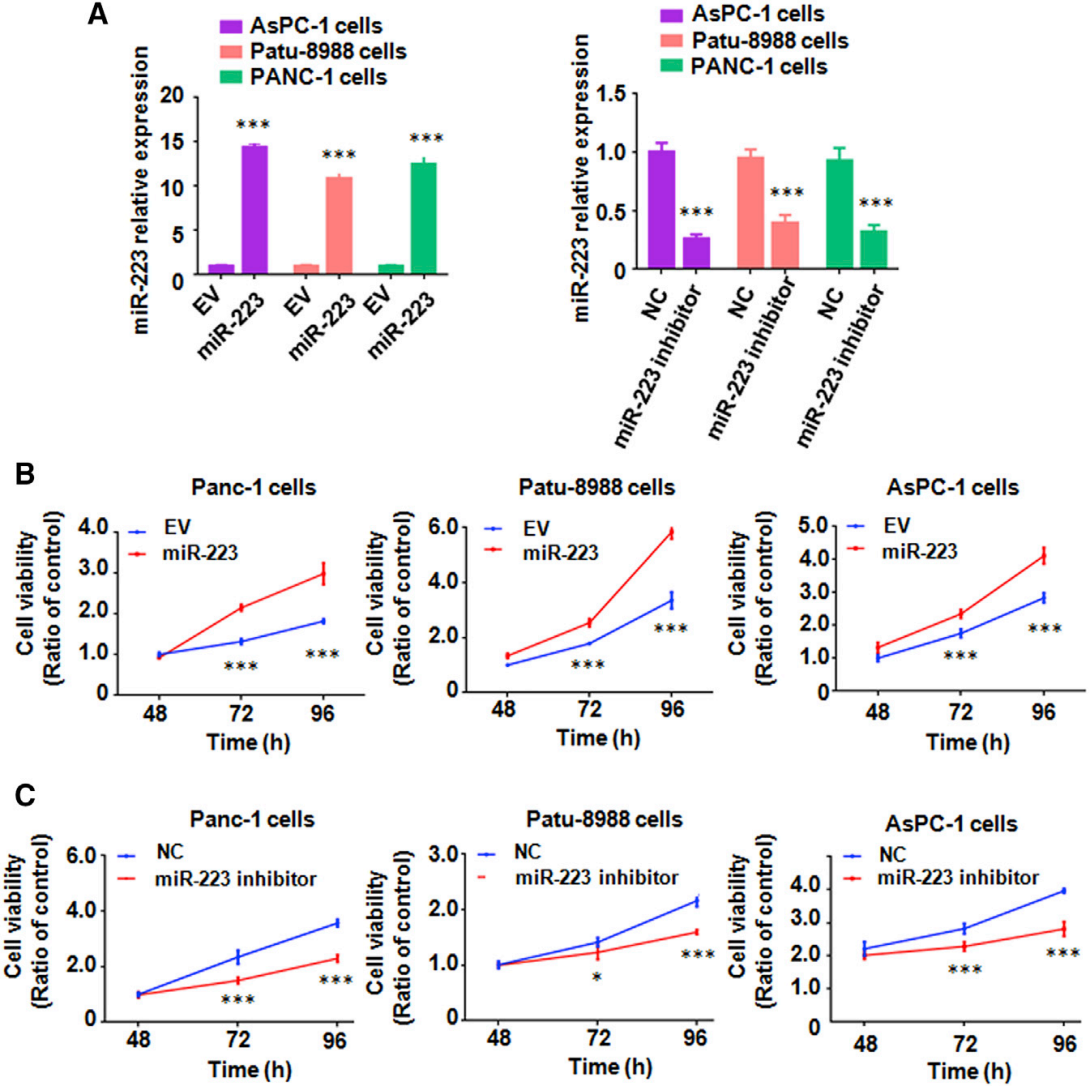
Overexpression of miR-223 Promotes Cell Growth in PC Cells (A) Real-time RT-PCR assay was conducted to detect the expression of miR-223 in PC cells after miR-223 overexpression or miR-223 inhibition. EV, empty vector; miR-223, miR-223 mimics; NC, nonspecific miRNA. ***p < 0.001 versus control. (B) MTT assay was conducted to measure the cell growth in PC cells after miR-223 overexpression for different times. ***p < 0.001 versus control. (C) MTT assay was conducted to detect the cell growth in PC cells after miR-223 inhibition for different times. ***p < 0.001 versus control.

**Figure 2:**
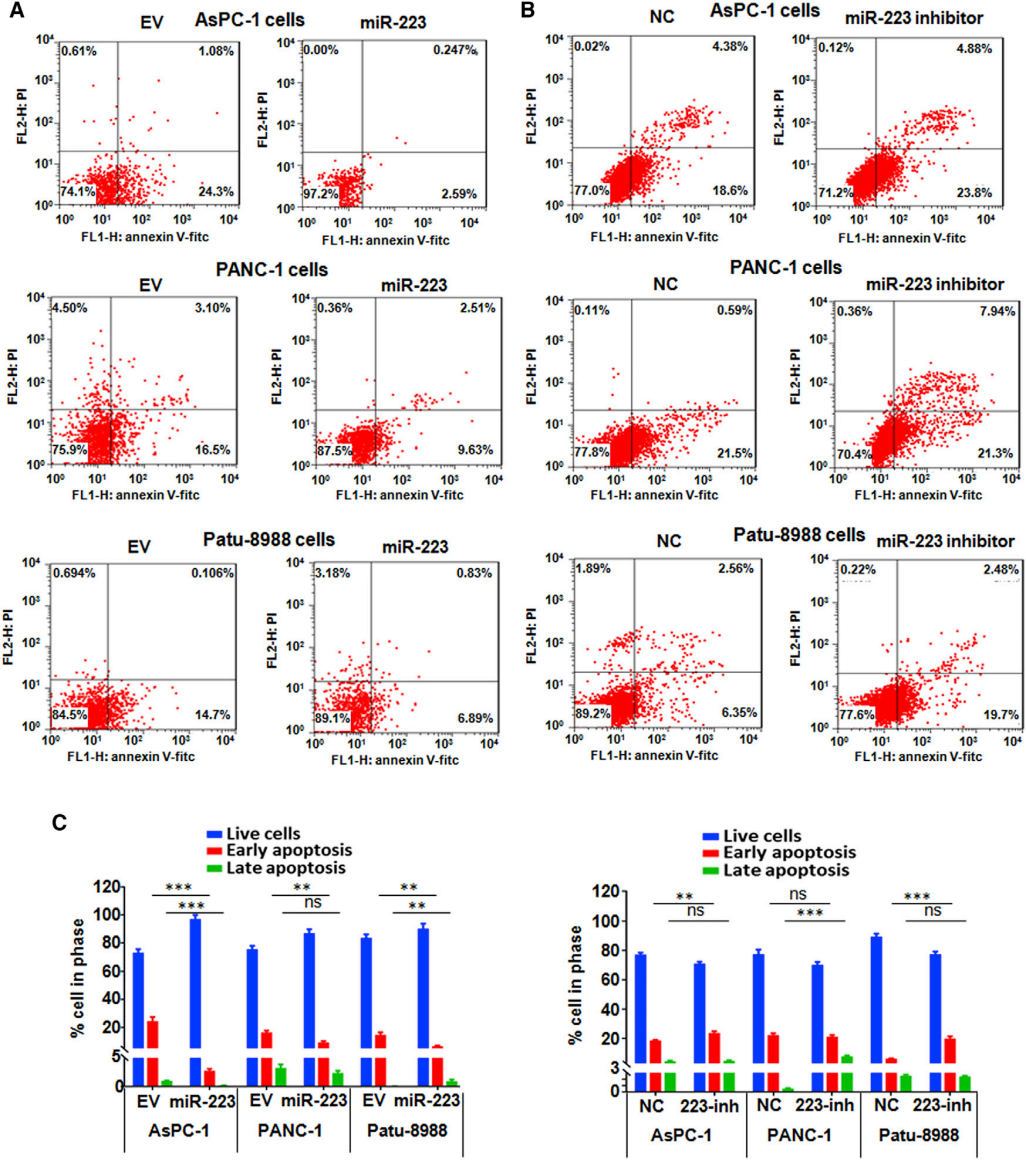
Overexpression of miR-223 Inhibits Cell Apoptosis in PC Cells (A) Apoptosis was measured in PC cells after miR-223 overexpression. EV, empty vector; miR-223, miR-223 mimics. (B) Apoptosis was detected in PC cells after miR-223 inhibition. NC, nonspecific miRNA. (C) Apoptosis was analyzed in PC cells after miR-223 overexpression or inhibition. **p < 0.01; ***p < 0.001 versus control. EV, empty vector; miR-223, miR-223 mimics; NC, nonspecific miRNA; 223-inh, miR-223 inhibitor; ns, no significant difference.

To explore the role of miR-223 in PC cell growth, an MTT assay was performed in PC cells after miR-223 mimic or miR-223 inhibitor treatments. We found that the miR-223 level was significantly increased after its mimic treatment (Figure 1A). We also observed that miR-223 level was decreased in PC cells with miR-223 inhibitor treatment (Figure 1A). Our MTT results showed that overexpression of miR-223 enhanced cell growth in PC cells (Figure 1B). In line with this finding, downregulation of miR-223 inhibited cell growth in PC cells (Figure 1C). Cell apoptosis assays revealed that overexpression of miR-223 suppressed cell apoptosis in PC cells (Figure 2A). Conversely, inhibition of miR-223 induced cell apoptotic death in PC cells (Figure 2B). Interestingly, we observed that miR-223 mainly regulated early apoptosis in PC cells (Figure 2C). These findings demonstrated that miR-223 plays an oncogenic role in PC cells.

#### Overexpression of miR-223 Enhances Cell Migratory and Invasive Activity

**Figure 3:**
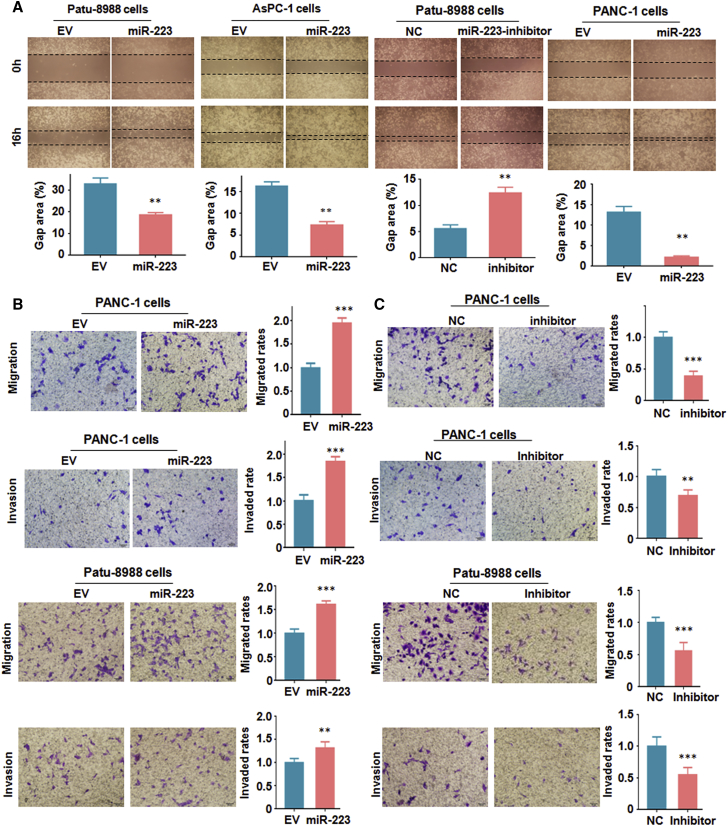
Overexpression of miR-223 Promotes Cell Migration and Invasion in PC Cells (A) Top: migration was measured by wound healing assay in PC cells after miR-223 overexpression or inhibition. Bottom: quantitative results are shown for data in the top panel. **p < 0.01 versus control. EV, empty vector; miR-223, miR-223 mimics; NC, nonspecific miRNA. (B) Left: migration and invasion assays were performed to measure the migrated capacity in PC cells after miR-223 overexpression. Right: quantitative results are shown for data in the left panel. ***p < 0.001 versus control. (C) Left: migration and invasion assays were conducted to measure the invasive capacity in PC cells after miR-223 inhibition. Right: quantitative results are shown for data in the left panel. ***p < 0.001 versus control.

To dissect the function of miR-223 in regulation of cell motility in PC cells, we performed the wound healing assay and Transwell assay in PC cells after treatment with miR-223 mimics or miR-223 inhibitors. We found that PC cells with overexpression of miR-223 have increased numbers of cells migrating across the wound (Figure 3A). However, PC cells with miR-223 inhibitor treatments reduced cell migration numbers compared with the control group (Figure 3A). In support of miR-223’s oncogenic role, our Transwell assay results showed that miR-223 mimic treatment enhanced cell migration and invasion in PC cells (Figure 3B). In line with this finding, treatment with miR-223 inhibitors retarded the cell migration and invasion in PC cells (Figure 3C). These results clearly suggest that miR-223 is involved in PC cell migration and invasion.

### PDS5B Is a Downstream Target of miR-223

**Figure 4:**
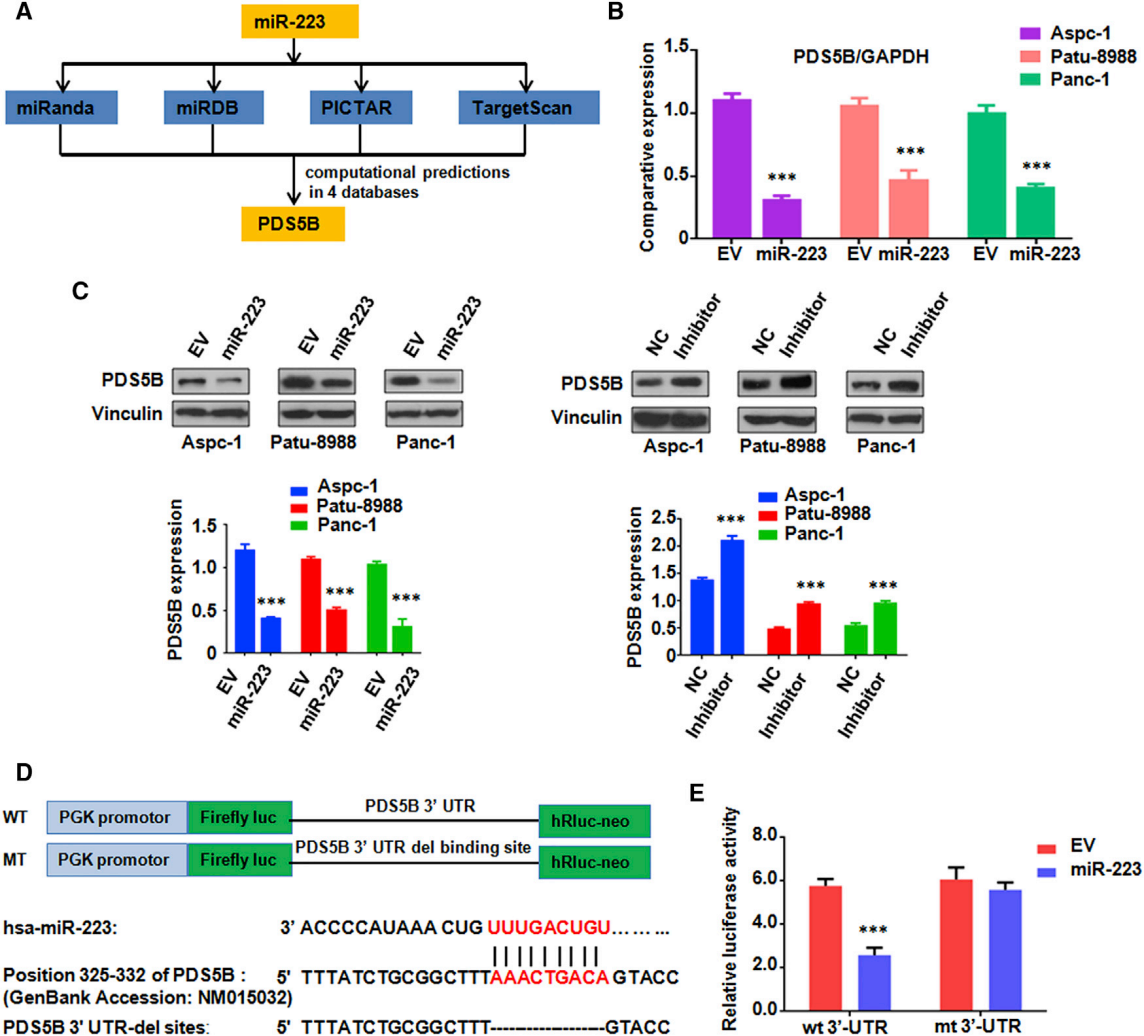
PDS5B Is a Target of miR-223 (A) Four public algorithms—miRanda, miRDB, PicTar, and TargetScan—were used to predict that PDS5B might be a potential target of miR-223. (B) Real-time RT-PCR assay was performed to detect the mRNA level of PDS5B in PC cells treated with miR-223 mimics. EV, empty vector; miR-223, miR-223 mimics. ***p < 0.001 versus control. (C) Top: western blotting analysis was used to measure protein level of PDS5B in PC cells treated with miR-223 mimics or inhibitor. Bottom: quantitative results are shown for the top panel. EV, empty vector; Inhibitor, miR-223 inhibitor. ***p < 0.001 versus control. (D) Sequences of wild-type and mutant target sites for miR-223 in PDS5B are shown. (E) Luciferase reporter assays were performed in 293T cells to identify the binding of miR-223 to PDS5B 3′ UTR. wt, wild-type; mt, mutation. ***p < 0.001 versus control.

According to the data from four public algorithms—TargetScan, PicTar, miRDB, and miRanda—PDS5B was identified to be a potential target of miR-223 (Figure 4A). To validate this hypothesis, we conducted real-time RT-PCR to measure the expression changes of PDS5B in PC cells after treatment with miR-223 mimics. Notably, we found that treatment with miR-223 mimics led to PDS5B downregulation in PC cells (Figure 4B). To further confirm our hypothesis, western blotting analysis was used to measure the protein level changes of PDS5B in PC cells treated with miR-223 mimics. We observed the downregulation of PDS5B in PC cells treated with miR-223 mimics (Figure 4C). Consistently, inhibition of miR-223 by its inhibitor increased the expression of PDS5B in PC cells (Figure 4C). Further bioinformatics analysis revealed that PDS5B 3′ UTR harbors potential miR-223 target sites (Figure 4D). To verify PDS5B as a potential target of miR-223, a reporter assay was conducted in cells with the luciferase gene driven by either wild-type or mutated PDS5B (Figure 4D). We found that it has a significant reduction in luciferase activity with wild-type PDS5B but not mutated PDS5B 3′ UTR in HEK293T cells transfected with miR-223 mimic (Figure 4E). Taken together, our results indicate that PDS5B is a downstream target of miR-223.

### PDS5B Overexpression Inhibits Cell Growth and Induces Cell Apoptosis

**Figure 5:**
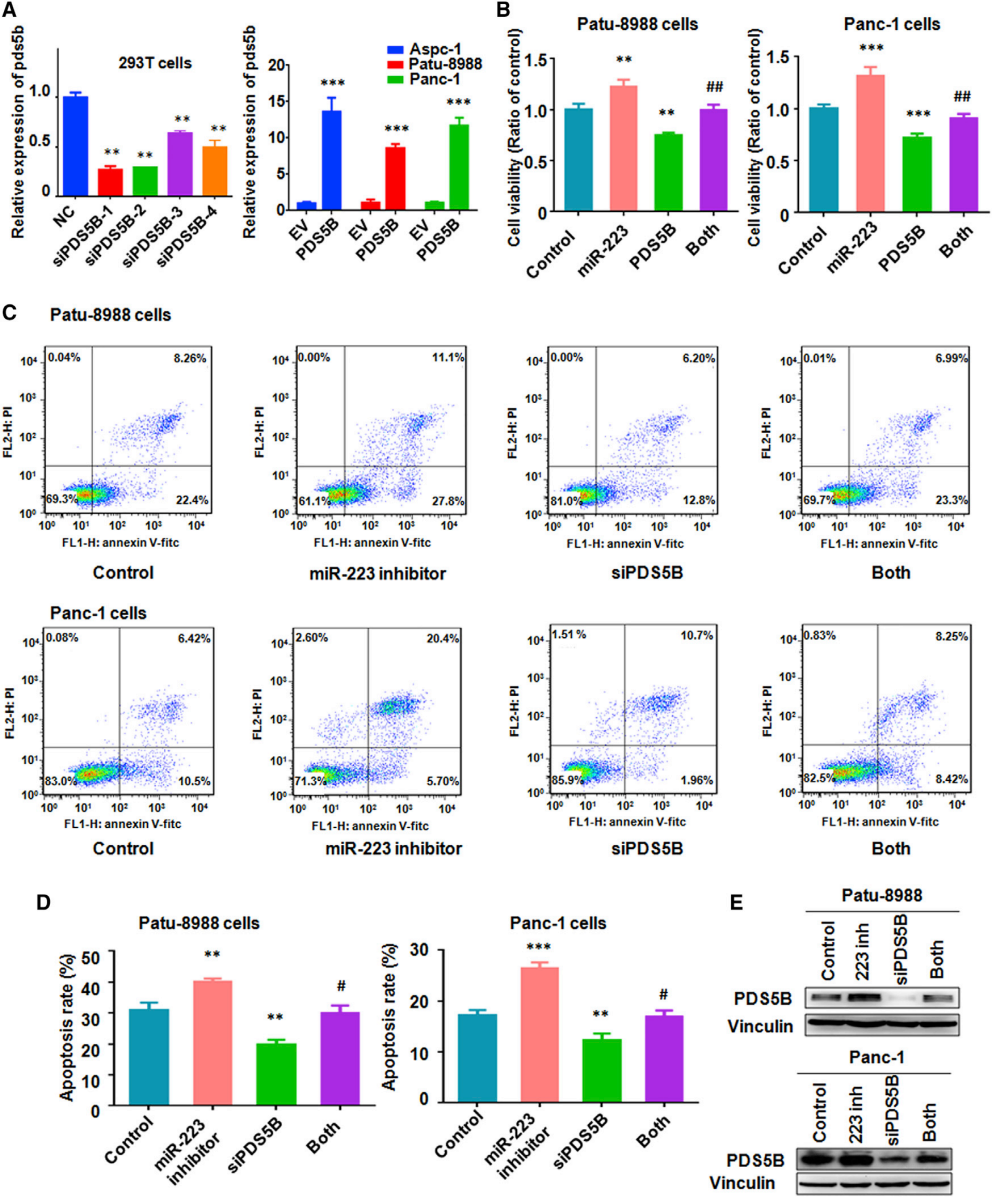
Overexpression of PDS5B Reverses miR-223-Mediated Cell Growth Promotion and Apoptosis Inhibition (A) Left: Real-time RT-PCR was used to measure the mRNA level of PDS5B in 293T cells after PDS5B siRNA transfection. Right: real-time RT-PCR was performed to measure PDS5B mRNA level in PC cells after PDS5B cDNA transfection. NC, nonspecific control siRNA; siPDS5B, PDS5B siRNA; EV, empty vector; PDS5B, PDS5B cDNA transfection. (B) MTT assay was used to measure the cell growth in PC cells after PDS5B overexpression plus miR-223 overexpression. Control, empty vector and nonspecific miRNA; miR-223, miR-223 mimics; PDS5B, PDS5B cDNA; Both, miR-223 mimics plus PDS5B cDNA transfection. **p < 0.01; ***p < 0.001 versus control; ^##^p < 0.01 versus miR-223 overexpression or PDS5B overexpression alone. (C and D) Apoptosis was measured in Patu-8988 (C) and Panc-1 (D) cells after PDS5B downregulation plus miR-223 inhibitor treatment. Control, nonspecific miRNA plus control siRNA; siPDS5B, PDS5B siRNA; Both, PDS5B siRNA plus miR-223 inhibitor. **p < 0.01; ***p < 0.001 versus control; ^#^p < 0.05 versus miR-223 inhibitor or PDS5B siRNA transfection alone. (E) Western blotting was used in PC cells after PDS5B downregulation plus miR-223 inhibitor treatment. Control, nonspecific miRNA plus control siRNA; 223 inh, miR-223 inhibitor; siPDS5B, PDS5B siRNA; Both, PDS5B siRNA plus miR-223 inhibitor.

To determine whether PDS5B controls cell growth, PDS5B small interfering RNAs (siRNAs) were used to downregulate the expression of PDS5B. Our real-time RT-PCR results also showed that PDS5B mRNA was downregulated by its siRNA (Figure 5A). Conversely, real-time RT-PCR data revealed that PDS5B was significantly increased by its cDNA transfection (Figure 5A). Next, an MTT assay was applied for measuring cell growth in PC cells after manipulating PDS5B by either PDS5B cDNA or siRNA transfections. Notably, we observed that overexpression of PDS5B inhibited cell growth in PC cells (Figure 5B). In keeping with this result, PDS5B downregulation inhibited cell apoptosis in PC cells (Figures 5C and 5D). Western blotting results also confirmed that PDS5B siRNA transfection reduced the level of PDS5B in PC cells (Figure 5E). Altogether, these results coherently support the notion that PDS5B could play a tumor-suppressive role in PC cells.

### PDS5B Downregulation Enhances Cell Motility

**Figure 6:**
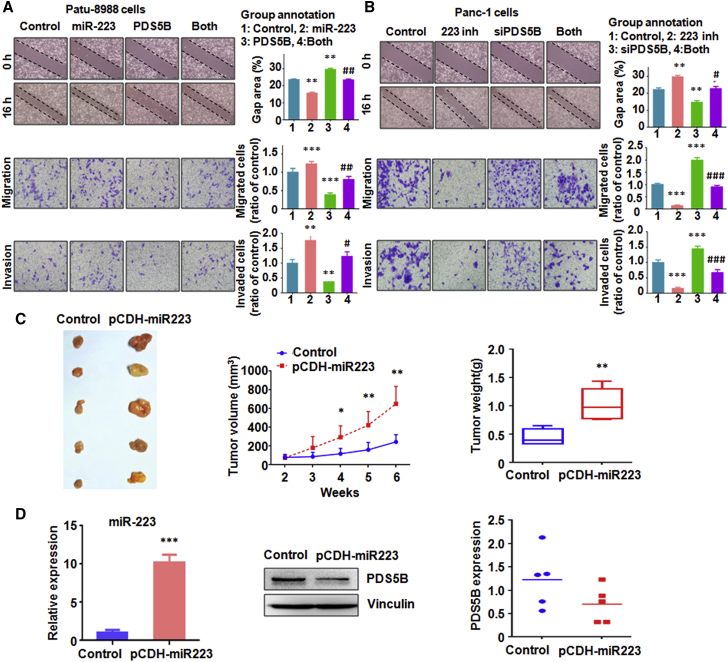
Overexpression of PDS5B Reverses miR-223-Mediated Cell Migration and Invasion (A) Top left: migration was measured by wound healing assay in PC cells after both miR-223 overexpression and PDS5B overexpression. Top right: quantitative results are shown for data in the top left panel. Bottom left: migration and invasion assays were performed to measure the migrated and invasive capacities in PC cells after both miR-223 overexpression and PDS5B overexpression. Bottom right: quantitative results are shown for data in the bottom left panels. Control, empty vector and nonspecific miRNA; miR-223, miR-223 mimics; PDS5B, PDS5B cDNA; Both, miR-223 mimics plus PDS5B cDNA transfection. **p < 0.01; ***p < 0.001 versus control; ^#^p < 0.05; ^##^p < 0.01 versus miR-223 overexpression alone or PDS5B overexpression alone. (B) Top left: migration was measured by wound healing assay in PC cells after both miR-223 inhibitor treatment and PDS5B inhibition. Top right: quantitative results are shown for data in the top left panel. Bottom left: migration and invasion assays were performed to measure the migrated and invasive capacities in PC cells after both miR-223 inhibitor treatment and PDS5B inhibition. Bottom right: quantitative results are shown for data in the bottom left panels. Control, nonspecific miRNA plus control siRNA; 223 inh, miR-223 inhibitor; siPDS5B, PDS5B siRNA; Both, PDS5B siRNA plus miR-223 inhibitor. **p < 0.01. ***p < 0.001 versus control; ^#^p < 0.05; ^###^p < 0.001 versus miR-223 inhibitor alone or PDS5B inhibition alone. (C) Left: overexpression of miR-223 enhanced tumor growth in mice. Middle: tumor volume was measured in mice. *p < 0.05; **p < 0.01 control. Right: tumor weight was measured in mice. **p < 0.01 control. (D) Left: real-time RT-PCR assay was used to measure the miR-223 in mouse tumor. Middle: western blotting was used to measure the PDS5B level in mouse tissues. Right: quantitative results of PDS5B protein level in mouse tissues.

To investigate whether PDS5B could regulate the cell motility, a wound healing assay was performed in PC cells after overexpression or downregulation of PDS5B in PC cells. The results from our wound healing assay showed that downregulation of PDS5B promoted cell migration in PC cells and that, conversely, PDS5B upregulation retarded cell migration (Figure 6A). Transwell assay results clearly demonstrated that PDS5B downregulation enhanced cell migration and invasion, whereas PDS5B overexpression suppressed cell invasion in PC cells (Figures 6A and 6B). Taken together, PDS5B controlled cell motility in PC cells.

### Overexpression of PDS5B Reverses miR-223-Mediated Tumor Progression

To validate whether PDS5B is critically involved in miR-223-triggered cell growth and invasion, the PC cells were transfected with PDS5B cDNA and treated with miR-223 mimics. We found that overexpression of PDS5B partly annulled miR-223-triggered cell growth promotion in PC cells (Figure 5B). Moreover, in support of PDS5B as the major downstream pathway through which miR-223 exerts its biological functions in governing apoptosis process, downregulation of PDS5B reversed miR-223-inhibitor-induced cell apoptosis in PC cells (Figures 5C and 5D). Furthermore, our wound healing assay results showed that PDS5B downregulation partly rescued miR-223-inhibitor-induced cell migration inhibition (Figure 6B). Similar results were observed in Transwell assay for cell migration and invasion (Figures 6A and 6B). Meanwhile, PDS5B overexpression rescued miR-223-enhanced cell migration and invasion in PC cells (Figures 6A and 6B).

### Overexpression of miR-223 Enhances Tumor Growth *In Vivo*

To assess the biological effects of miR-223 in PC *in vivo*, nude mice were injected subcutaneously with PaTu-8988 cells with lentivirus stable expression of pre-miR-223. We observed that elevation of miR-223 clearly promoted subcutaneous tumor growth compared with that in the control group (Figure 6C). We also investigated the expression of Pds5B and the miR-223 level. We found that the expression of miR-223 in tumors was increased but that the expression of PDS5B was decreased in tumors (Figure 6D). These data indicated that miR-223 enhances tumor growth in part via downregulation of PDS5B.

## Discussion

Accumulated evidence has demonstrated that miR-223 plays an oncogenic role in PC development and progression.^17^, ^22^ In line with this note, the level of miR-223 was increased in PC tumors compared with normal-appearing adjacent tissues.^27^ A similar result was found in another study, showing that miR-223 expression was significantly higher in PC tissues.^22^ Moreover, miR-223 was increased in a genetically engineered KrasG12D; Pdx1-Cre mouse, which is a good model representing PC development and progression.^28^ Notably, miR-223 could be a potential biomarker in whole blood for the detection of PC.^17^ Another study further supported that plasma miR-223 might be a clinically useful biomarker for screening PC and predicting the invasiveness of PC.^22^ Recently, it has been reported that the HnRNPK/miR-223/FBXW7 feedback cascade promoted PC cell growth and invasion.^16^ These reports analyze whether miR-223 plays an important role in PC development and progression. In the present study, we reported that miR-223 targeted PDS5B in PC, leading to the enhancement of cell growth and invasion. Our results provide a new mechanism of miR-223-triggered PC progression.

PDS5B expression levels were reported to be associated with histological grade in breast cancer.^29^ Moreover, PDS5B could be a predictor of outcome after chemotherapy in breast cancer.^29^ Another study demonstrated that low levels of PDS5B correlated with better survival in high-grade serous epithelial ovarian cancer patients.^25^ Similarly, low PDS5B was correlated with longer overall survival of gastrointestinal stromal tumors.^30^ Additionally, loss of PDS5B was found to be a feature of gastric and colorectal cancers with high microsatellite instability.^26^ Notably, loss of PDS5B disrupted stem cell programs in embryonal carcinoma as a new mechanism of PDS5B in tumor suppression.^31^ Zhou et al. reported that overexpression of PDS5B inhibited proliferation and promoted apoptosis in embryonal carcinoma cells.^32^ Interestingly, PDS5B was highly expressed in oral squamous cell carcinoma, suggesting that the function of PDS5B could be cell-type dependent.^33^ Since the role of PDS5B in PC is poorly understood, we explored the function of PDS5B in PC cells. We found that PDS5B overexpression inhibited cell growth and invasion, whereas downregulation of PDS5B promoted cell motility, suggesting that PDS5B could be a tumor suppressor in PC. Our work helps to understand how PDS5B is regulated in PC.

Taken together, we reported that the miR-223/PDS5B axis is important for the PC progression. Targeting this axis could be a useful approach for treating PC. Our previous studies showed that downregulation of miR-223 reversed the epithelial-mesenchymal transition in gemcitabine-resistant PC cells.^34^ Moreover, genistein decreased miR-223 expression in PC cells.^35^ In addition, we found a synergistic reversal effect of epithelial-mesenchymal transition (EMT) by miR-223 inhibitor and genistein in gemcitabine-resistant PC cells.^36^ Therefore, genistein inhibited PC progression in part via the downregulation of miR-223 and upregulation of PDS5B, which need to be validated. In summary, our findings revealed a new molecular mechanism of miR-223-mediated tumor progression in PC cells. This study further indicated that inactivation of miR-223 or upregulation of PDS5B could be a novel strategy for treating PC.

## Materials and Methods

### Cell Culture, Reagents, and Antibodies

The human pancreatic AsPC-1 cells were cultured in RPMI 1600 (Invitrogen, Carlsbad, CA, USA), and PaTu-8988 and PANC-1 cells were cultured in DMEM (GIBCO, Gaithersburg, MD, USA) supplemented with 10% fetal bovine serum (FBS) in 5% CO_2_ at 37°C. MTT (3-(4,5-dimethythiazol-2-yl)-2,5-diphenyl tetrazolium bromide) was purchased from Sigma (St. Louis, MO, USA). Antibodies against PDS5B and vinculin were purchased from Bethyl Laboratories (Montgomery, TX, USA). The secondary antibodies were obtained from Cell Signaling Technology. Transwell inserts and Matrigel were purchased from BD Biosciences. The TaqMan miRNA assay was purchased from Applied Biosystems (Foster City, CA, USA).

### MTT Assays

The PC cells were seeded into 96-well plates (5 × 10^3^ cells per well) for overnight incubation. Then, cells were treated with miR-223 mimics or miR-223 inhibitor for different times. An MTT assay was performed as described previously.^37^

### Cell Apoptosis Assay

The PC cells were treated with different transfections. Then, the cells were collected and subjected to Annexin V-FITC (fluorescein isothiocyanate)/propidium iodide (PI) staining, following the manufacturer’s instructions. The cell apoptosis was analyzed by flow cytometry as described previously.^36^

### Transwell Migration and Invasion Assays

The Transwell migration and invasion assays were performed using a 24-well plate with 8-mm-pore-size chamber inserts (Corning, New York, NY, USA) as described previously.^38^ Briefly, the invasion assay used Transwell inserts precoated with Matrigel, while the migration assay used non-coated inserts. Cells were seeded into the upper chamber of the insert, which was suspended in serum-free culture medium. The lower chamber was filled with medium with 10% FBS. After incubation for 20–24 h, we used cotton swabs to scrape the cells in the upper chamber. The migrated and invaded cells on the bottom surface of chambers were fixed with 4% paraformaldehyde for 20 min and subsequently stained with Giemsa solution. The stained cells were imaged and counted in 5 fields with random choice.

### Wound Healing Assays

The PC cells were seeded in 6-well plates. After the cells reached more than 95% confluence, the wounded scrape was made by a pipette tip. The generated floating cells were removed using PBS. The wound healing was photographed at 0 and 16 h as described previously.^39^

### Real-Time RT-PCR

Total RNA was obtained using TRIzol Reagent (Invitrogen, CA, USA) following the manufacturer’s protocol. The miR-223 expression was analyzed using the TaqMan miRNA assays according to the manufacturer’s instructions. The expression level of miR-223 was normalized using U6 small nuclear RNA (snRNA) levels. The expression of PDS5B was determined using the SYBR Green RT-PCR Assay (Takara, Dalian, China) and normalized to GAPDH as previously described.^40^ The primers used in the PCR reaction are as follows: PDS5B, forward primer, 5′ TCC ACA CAG TCC ACA CCA C-3′, and reverse primer, 5′-ATC ATT TTC CTT AGT AGC TGC-3′; GAPDH, forward primer, 5′-CAG CCT CAA GAT CAG CA-3′, and reverse primer, 5′-TGT GGT CAT GAG TCC TTC CA-3′.

### Western Blotting Analyses

The cells were washed twice with PBS and lysed with RIPA buffer supplemented with protease inhibitors. The protein concentrations were detected using a bicinchoninic acid (BCA) protein assay. The proteins were separated using SDS-PAGE and transferred to polyvinylidene fluoride (PVDF) membranes. The membranes were then blocked with 5% nonfat milk and immunoblotted with antibodies as described earlier.^41^

### Prediction of miR-223 Target

To explore the molecular mechanism of miR-223-regulated cell growth and invasion in PC cells, we sought to identify the downstream target of miR-223. The data from four public algorithms—TargetScan, PicTar, miRDB, and miRanda—were used to identify the potential target of miR-223.

### miRNA-223 Inhibitor and Mimic Transfection

The cells were seeded in six-well plates and transfected with miR-223 inhibitor or the nonspecific control or miR-223 mimics (GenePharma, Shanghai, China) using DharmaFect Transfection Reagent (Dharmacon, Lafayette, CO, USA) following the manufacturer’s protocol.^39^ The miR-223 inhibitor was: 5′-UGG GGU AUU UGA CAA ACU GAC A-3′. The cells were subjected to further analysis as described in the Results section.

### *In Vivo* Animal Experiments

Five-week-old male nude mice were subcutaneously inoculated with 1 × 10^7^ PaTu-8988 cells after miR-223 treatment. The tumor size was measured with a caliper every 5 days. Tumor volume was calculated as (length × width × height)/2. Mice of each group were killed after 3 weeks of injections, and the xenografted tumors were excised and examined by western blotting. All animal studies and procedures were approved by the Institutional Animal Care and Use Committee.

#### Luciferase Assays

The wild-type and mutant PDS5B 3′ UTR were amplified by PCR and cloned in pmirGLO vector (Promega) with firefly luciferase. A total of 5 × 10^4^ cells treated with control miR-223 mimics were transfected with wild-type or mutants of PDS5B 3′ UTR luciferase reporters. After 48 h of transfection, the firefly luciferase and Renilla luciferase were measured according to the manufacturer’s protocols (Promega, Madison, WI, USA). The firefly luciferase activities were normalized to Renilla luciferase activities.

### Statistical Analyses

Statistical comparisons were analyzed by Student’s t test for comparing two groups, using one-way ANOVA, followed by Tukey’s post hoc test for comparing multiple groups, using GraphPad Prism 4.0 (GraphPad Software, La Jolla, CA) in cell growth, migration, and invasion. The error bars represent SD. p < 0.05 was considered statistically significant.

## Author Contributions

J.M. conceived the work, designed and performed the experiments, analyzed the data, and wrote the manuscript. T.C., Y.C., and F.Z. designed and performed the experiments and analyzed the data. Y.S. collected animal tissues and analyzed the data. Z.P.W. and J.X. conceived the work, wrote the manuscript, and critically viewed and supervised the study.

## Conflicts of Interest

The authors declare no conflict of interest.
